# Supplementary material for: Tailoring dengue health communication: Survey-based strategies to reduce message fatigue across risk areas
Source: PLoS Negl Trop Dis. 2025 May 30;19(5):e0012723. doi: 10.1371/journal.pntd.0012723 (PMC12148230; doi:10.1371/journal.pntd.0012723)
Supplement: S1 Table — (PDF) [file pntd.0012723.s001.pdf]

您好，這是一份關於登革熱的線上問卷調查，能否麻煩您花幾分鐘協助填答，答案沒有對錯可言，請您就自己的想法回答即可，謝謝您！為感謝您撥冗參加研究，再送出本問卷後，您可選擇在最後填寫您的電子信箱，以參加抽獎活動。獎品為統一超商禮券 200 元 (5 名)，我們將以電子郵件方式聯繫得獎者並發送獎勵。重複提交不會增加獲獎機會，且可能會導致取消資格。

國立中正大學傳播學系教授盧鴻毅
國立台灣師範大學健康促進與衛生教育學系專案助理教授林佳嫻
敬上

I、首先，想請教您對台灣目前登革熱疫情的感受如何？答案從「一點也不嚴重」（0）到「非常嚴重」（10），數字愈大代表愈嚴重，請您就自己的感受挑選一個最適合的數字。

（1）您認為當前台灣登革熱疫情嚴不嚴重？

0---- 1 ---- 2 ---- 3 ---- 4 ---- 5 ---- 6 ---- 7 ---- 8 ---- 9 ---- 10

（2）您認為一旦染上登革熱，引發的死亡機率很大?

0---- 1 ---- 2 ---- 3 ---- 4 ---- 5 ---- 6 ---- 7 ---- 8 ---- 9 ---- 10

II、請問您覺得「自己」或「身邊其他人」感染登革熱的可能性如何？答案從「非常不可能」（**0**）到「非常可能」（**10**），數字愈大代表可能性愈高，請您就自己的感受挑選一個最適合的數字。

（1）您認為自己感染登革熱的可能性如何？

0---- 1 ---- 2 ---- 3 ---- 4 ---- 5 ---- 6 ---- 7 ---- 8 ---- 9 ---- 10

（2）您認為身邊其他人，感染登革熱的可能性如何？

0---- 1 ---- 2 ---- 3 ---- 4 ---- 5 ---- 6 ---- 7 ---- 8 ---- 9 ---- 10

III、想瞭解您平常對於登革熱新聞的關注程度為何？以及對您看完或聽完相關新聞之後是否會進一步思考？請您就個人的狀況，針對每一句陳述，挑選一個最適合的答案。

	(1) 非常不同意	(2) 不同意	(3) 有點不同意	(4) 沒意見	(5) 有點同意	(6) 同意	(7) 非常同意
1. 我最近會一直關注「報紙」上有關登革熱的新聞							
2. 我最近會一直關注「電視」上有關登革熱的新聞							
3. 我最近會一直關注「廣播」上有關登革熱的新聞							
4. 我最近會一直關注「網路」上有關登革熱的新聞							
5. 在看過或聽過登革熱的媒體新聞報導後，我會思考登革熱所造成的後果							
6. 我通常會將在媒體上看到或聽到的登革熱相關資訊與自身的生活經驗進行連結							
7. 當生活中遇到登革熱的相關討論時，我會想起並思考與登革熱有關的媒體報導內容							

IV、針對登革熱的相關新聞報導，請問您對各種媒體的報導內容可信度評價如何？請針對以下的敘述，分別挑選一個適合的答案。

	(1) 非常不同意	(2) 不同意	(3) 有點不同意	(4) 沒意見	(5) 有點同意	(6) 同意	(7) 非常同意
1. 我認為「報紙」上有關登革熱的報導值得相信							
2. 我認為「電視」上有關登革熱的報導值得相信							
3. 我認為「廣播」上有關登革熱的報導值得相信							
4. 我認為「網路」上有關登革熱的報導值得相信							

V、以下想了解您平常會不會主動尋求跟登革熱有關的資訊？請針對以下的敘述，分別挑選一個適合的答案。

	(1)非常不同意	(2)不同意	(3)有點不同意	(4)沒意見	(5)有點同意	(6)同意	(7)非常同意
1. 我會主動搜尋登革熱疫情狀況的相關資訊							
2. 我會主動搜尋登革熱感染途徑的相關資訊							
3. 我會主動搜尋感染登革熱後可能出現那些症狀的資訊							
4. 我會主動搜尋防治登革熱的相關資訊							
5. 我會主動搜尋治療登革熱的相關資訊							

VI、接下來想請教的是您對於當前登革熱疫情相關資訊的感受如何？答案從「非常不同意」到「非常同意」，請您針對每一句陳述，就自己的看法，挑選一個最合適的答案。

	(1)非常不同意	(2)不同意	(3)有點不同意	(4)沒意見	(5)有點同意	(6)同意	(7)非常同意
1. 這個社會一直在重複提醒我們登革熱是個嚴重的問題							
2. 在這當下，有關登革熱的資訊已經超出我的需要							
3. 有關登革熱的相關資訊，我已經聽到不想聽							
4. 登革熱的相關訊息實在是多到爆表							
5. 登革熱的相關資訊都在炒冷飯，了無新意							
6. 我都可以猜出接下來有關登革熱的相關資訊會是那些							
7. 我已經受夠了登革熱的相關資訊							
8. 我對於登革熱會造成那些後果的相關資訊感到厭煩							
9 看到或聽到登革熱的相關資訊，我只想大嘆一口氣							
10. 提到登革熱的相關資訊，我不禁要打個哈欠							
11. 我覺得當前登革熱的相關資訊單調無趣							

VII，以下幾道題目想了解您如何看待政府處理登革熱疫情的能力，答案從「非常不同意」到「非常同意」，請您就自己的看法，挑選一個最合適的答案。

	(1)<br>非<br>常<br>不<br>同<br>意	(2)<br>不<br>同<br>意	(3)<br>有<br>點<br>不<br>同<br>意	(4)<br>沒<br>意<br>見	(5)<br>有<br>點<br>同<br>意	(6)<br>同<br>意	(7)<br>非<br>常<br>同<br>意
1.我認為政府針對登革熱疫情所採取的防治政策是正確的							
2.我覺得政府正努力制訂長遠的登革熱疫情解決方案							
3.我認為政府有能力解決當前的登革熱疫情							

VIII、最後想請教的是您會針對登革熱採取那些預防行為？答案從「非常不同意」到「非常同意」，請您就自己的看法，挑選一個最合適的答案。

	(1)<br>非<br>常<br>不<br>同<br>意	(2)<br>不<br>同<br>意	(3)<br>有<br>點<br>不<br>同<br>意	(4)<br>沒<br>意<br>見	(5)<br>有<br>點<br>同<br>意	(6)<br>同<br>意	(7)<br>非<br>常<br>同<br>意
1. 如果從事戶外活動，我會穿著淺色長袖衣褲							
2. 皮膚裸露處，我會使用防蚊液（乳）							
3. 我會仔細巡視家戶內外積水容器，並將積水倒掉							
4. 家中不要的容器，我會將它清除							
5. 留下的器物，我會刷洗以去除斑蚊蟲卵，並妥善收拾或予以倒置							
6. 家中會儘量加裝紗窗以防蚊子進入							
7. 我會儘量減少前往登革熱疫區活動的機會							

（一）基本資料

1.性別：

□（1）男性　□（2）女性

2.目前的居住地

□（1）北（臺北市、新北市、基隆市、桃園市、新竹市、新竹縣、宜蘭縣）

□（2）中（苗栗縣、臺中市、彰化縣、南投縣、雲林縣）

□（3）南（嘉義市、嘉義縣、臺南市、高雄市、屏東縣）

□（4）東（花蓮縣、臺東縣）

□（5）離島（金門縣、連江縣、澎湖縣、連江縣）

3.年齡 (實歲)

__________ 歲

4.教育程度

□（1）國小以下　□（2）國小　□（3）國中　□（4）高中（職）

□（5）大學（專）　□（6）研究所（碩、博士）

5.平均家庭月收入：

□（1）0 ~ 2,0000　□（2）20,001 ~ 40,000　□（3）40,001~ 60,000

□（4）60,001 ~ 80,000　□（5）80,001 ~ 100,000　□（6）100,001 以上

6.您的個人政黨傾向

□（1）國民黨　□（2）民進黨　□（3）民眾黨　□（4）時代力量

□（5）親民黨　□（6）基進黨　□（7）沒有

7. 您從事的工作與登革熱防治有沒有關係？

□（1）有　□（2）沒有

Hello, this is an online questionnaire about dengue. May we kindly ask you to take a few minutes to help us complete this survey?

There are no right or wrong answers—please respond according to your own opinions. Thank you!

As a token of appreciation for your participation, you may provide your email address at the end of the survey to enter a prize draw.

The prize consists of five NT$200 7-Eleven gift cards. Winners will be contacted via email and rewarded accordingly.

Submitting the survey multiple times will not increase your chances and may lead to disqualification.

Professor Hung-Yi Lu, Department of Communication, National Chung Cheng University

Assistant Professor Chia-Hsien Lin, , Department of Health Promotion and Health Education, National Taiwan Normal University

I. Perception of Current Dengue Situation in Taiwan

1. Do you think the current dengue outbreak in Taiwan is severe? (0 = Not serious at all, 10 = Very serious)

0---- 1 ---- 2 ---- 3 ---- 4 ---- 5 ---- 6 ---- 7 ---- 8 ---- 9 ---- 10

2. Do you think the likelihood of death is high once infected with dengue? (0 = Very unlikely, 10 = Very likely)

0---- 1 ---- 2 ---- 3 ---- 4 ---- 5 ---- 6 ---- 7 ---- 8 ---- 9 ---- 10

II. Perceived Risk of Infection

1. How likely do you think it is that you will get a dengue infection? (0 = Very unlikely, 10 = Very likely)

0---- 1 ---- 2 ---- 3 ---- 4 ---- 5 ---- 6 ---- 7 ---- 8 ---- 9 ---- 10

2. How likely do you think it is that others around you will get a dengue infection? (0 = Very unlikely, 10 = Very likely)

0---- 1 ---- 2 ---- 3 ---- 4 ---- 5 ---- 6 ---- 7 ---- 8 ---- 9 ---- 10

III. Attention to Dengue News

We would like to understand how much attention you usually pay to news about dengue fever, and whether you reflect further after reading or hearing such news.

Please choose the most appropriate response for each statement based on your personal situation.

	(1) Strongly Disagree	(2) Disagree	(3) Somewhat Disagree	(4) Neutral	(5) Somewhat Agree	(6) Agree	(7) Strongly Agree
1. I follow dengue news in newspapers.							
2. I follow dengue news on TV.							
3. I follow dengue news on the radio.							
4. I follow dengue news online.							
5. I reflect on the consequences after seeing/hearing dengue news.							
6. I relate dengue news to my own life experiences.							
7. I recall and reflect on dengue news during related conversations.							

IV. Credibility of Dengue News in Media

Regarding news reports about dengue fever, how would you rate the credibility of the content across different types of media?

Please select the most appropriate response for each of the following statements.

	(1) Strongly Disagree	(2) Disagree	(3) Somewhat Disagree	(4) Neutral	(5) Somewhat Agree	(6) Agree	(7) Strongly Agree
1. I trust dengue news in newspapers.							
2. I trust dengue news on TV.							
3. I trust dengue news on the radio.							
4. I trust dengue news online.							

V. Information-Seeking Behavior

We would like to know whether you usually take the initiative to seek information related to dengue.

Please select the most appropriate response for each of the following statements.

	(1) Strongly Disagree	(2) Disagree	(3) Somewhat Disagree	(4) Neutral	(5) Somewhat Agree	(6) Agree	(7) Strongly Agree
1. I actively search for information about the current dengue outbreak situation.							
2. I actively search for information about how dengue is transmitted.							
3. I actively search for information							

about the possible symptoms after being infected with dengue.							
4. I actively search for information on how to prevent and control dengue.							
5. I actively search for information on how to treat dengue.							

VI. Perception of Dengue Information Overload

Next, we would like to ask how you feel about the current information related to the dengue outbreak. For each of the following statements, please select the response that best reflects your opinion, ranging from 'Strongly Disagree' to 'Strongly Agree'.

	(1) Strongly Disagree	(2) Disagree	(3) Somewhat Disagree	(4) Neutral	(5) Somewhat Agree	(6) Agree	(7) Strongly Agree
1. The seriousness of dengue is overtaught in our society.							
2. At this point, I’ve heard about problems related to dengue fever more than I ever needed to.							
3. I have heard enough about how serious dengue fever is.							
4. There are simply too many health							

messages about dengue fever nowadays.							
5. Dengue-related messages rarely provide new information.							
6. I can predict what a message about dengue fever is going to say.							
7. I am tired of hearing about the importance of preventing dengue.							
8. I am sick of hearing about the consequences of dengue.							
9. Dengue-related messages make me want to sigh.							
10. Dengue-related messages make me want to yawn.							
11. I find messages about dengue fever to be dull and monotonous.							

VII. Perceptions of Government Response

The following questions aim to understand your views on the government's ability to handle the dengue outbreak. For each statement, please select the response that best reflects your opinion, ranging from 'Strongly Disagree' to 'Strongly Agree'.

	(1) Strongly Disagree	(2) Disagree	(3) Somewhat Disagree	(4) Neutral	(5) Somewhat Agree	(6) Agree	(7) Strongly Agree
1. I believe the government's dengue prevention and control policies are appropriate.							
2. I feel the government is making efforts to develop long-term solutions for the dengue outbreak.							
3. I believe the government is capable of resolving the current dengue outbreak.							

VIII. Personal Preventive Behaviors

Lastly, we would like to ask what preventive actions you take against dengue fever. For each statement, please select the response that best reflects your opinion, ranging from 'Strongly Disagree' to 'Strongly Agree'.

	(1) Strongly Disagree	(2) Disagree	(3) Somewhat Disagree	(4) Neutral	(5) Somewhat Agree	(6) Agree	(7) Strongly Agree
1. When engaging in outdoor activities, I wear light-colored long-sleeved shirts and long							

pants.							
2. I apply mosquito repellent to exposed skin.							
3. I carefully inspect and empty containers with standing water in and around my home.							
4. I remove unused containers from my home.							
5. I scrub containers to remove Aedes mosquito eggs and store or turn them over properly.							
6. I try to install window screens at home to prevent mosquitoes from entering.							
7. I try to avoid visiting areas with dengue outbreaks.							

Demographic Information

1. Gender: □ Male □ Female

2. Residence: □ North □ Central □ South □ East □ Islands

3. Age (in years): ________

4. Education: □ Below Elementary □ Elementary □ Junior High □ High School □ University □ Graduate School

5. Monthly Household Income: □ 0–20,000 □ 20,001–40,000 □ 40,001–60,000 □ 60,001–80,000 □ 80,001–100,000 □ Over 100,001

6. Political Affiliation: □ KMT □ DPP □ TPP □ NPP □ PFP □ Taiwan State building Party □ None

7. Is your job related to dengue prevention? □ Yes □ No
