# Supplementary material for: Tailoring dengue health communication: Survey-based strategies to reduce message fatigue across risk areas
Source: PLoS Negl Trop Dis. 2025 May 30;19(5):e0012723. doi: 10.1371/journal.pntd.0012723 (PMC12148230; doi:10.1371/journal.pntd.0012723)
Supplement: S2 Table — Questions adapted from So et al. [13]. (PDF) [file pntd.0012723.s002.pdf]

S5 Table. Variables, codes and categories in the dataset

Variable	Code	Category/ Explanation
Sex	1	Male
	2	Female (reference group)
Resident	1	North (Low risk)
	2	Central (Low risk)
	3	South (High risk)
	4	Eastern (Low risk)
	5	Outlying island (Low risk)
Education	H	Higher than high school and equal to (reference group)
	L	Below high school
Job	1	Relevant job experience
	2	No job experience in dengue control (reference group)
Age		>=18
Perceived prevalence	0 – 10	Do you think the current dengue outbreak in Taiwan is severe?
Perceived severity	0 – 10	Do you think the likelihood of death is high once infected with dengue?
op_self	0 – 10	How likely do you think it is that you will get a dengue infection?
op_other	0 – 10	How likely do you think it is that others around you will get a dengue infection?
Optimistic bias	-10 – 10	op_self- op_other
ME 1 – 5	1 – 7	Questions in S2 Table. Message environment
ME	5 – 35	ME1 + ME2 + ME3 + ME4 + ME5
AR 1 – 4	1 – 7	Questions in S2 Table. Audience responses
AR	4 – 28	AR1 + AR2 + AR3 + AR4
