# Supplementary material for: Tailoring dengue health communication: Survey-based strategies to reduce message fatigue across risk areas
Source: PLoS Negl Trop Dis. 2025 May 30;19(5):e0012723. doi: 10.1371/journal.pntd.0012723 (PMC12148230; doi:10.1371/journal.pntd.0012723)
Supplement: S4 Table — (PDF) [file pntd.0012723.s004.pdf]

S4 Table. Multicollinearity assessments of independent variables in each risk area

Area	High risk			Low risk		
	GVIF	Df	Adjusted GVIF*	GVIF	Df	Adjusted GVIF*
Age	1.162	1	1.078	1.092	1	1.045
Sex	1.030	1	1.015	1.065	1	1.032
Education	1.190	1	1.091	1.079	1	1.039
Job	1.032	1	1.016	1.045	1	1.022
Perceived prevalence	1.536	1	1.239	1.370	1	1.170
Perceived severity	1.590	1	1.261	1.371	1	1.171
Optimistic bias	1.049	2	1.012	1.044	2	1.011

*GVIF^(1/(2*Df))
