# Supplementary material for: Tailoring dengue health communication: Survey-based strategies to reduce message fatigue across risk areas
Source: PLoS Negl Trop Dis. 2025 May 30;19(5):e0012723. doi: 10.1371/journal.pntd.0012723 (PMC12148230; doi:10.1371/journal.pntd.0012723)
Supplement: Attachment — Submitted filename: Comments to Authors-PNTD-D-24-01722.pdf [file pntd.0012723.s007.pdf]

Title: Different predictors of message fatigue across diverse risk areas for dengue prevention in Taiwan

Manuscript number: PNTD-D-24-01722

Comments to the authors:

This manuscript identified distinct predictors of message fatigue across different dengue-risk areas in Taiwan, including high, medium and low risk areas using survey data. The survey count data for message environment (ME) and audience response (AR) was analysed using both Poisson and negative binomial models. All negative binomial models were selected as the final output reporting only significant factors in this manuscript.

I would like to acknowledge the considerable effort that has gone into this manuscript. Understanding different factors that play a role in message fatigue including demographic factors and perception risks is highly important. As the authors state in the introduction, the knowledge of message fatigue for dengue risk in Taiwan is sparse. These findings may aid in informing updated prevention messages with future targeted implementation research specific to high, medium and low risk areas to encourage public compliance.

As the statistical analysis forms the major component of this study, the analysis should be further technically expanded in the materials and methods section to understand how the Likert scale data was treated. Also, displaying the distribution of the Likert scale data for each risk area using Likert plots or tables would greatly aid in improving this study. The Likert plots or tables would allow readers to clearly understand the overall distribution of the participants responses for message fatigue and perceived risk prior to the statistical analysis. Expanding on the above will significantly strengthen this manuscript. The major and minor comments are below.

Major comments:

Methods:

1. Was the study conducted and reported in compliance with the Consensus -Based Checklist for Reporting of Survey Studies (CROSS) guidelines? [1]. This checklist should be completed and added to the Supplementary Information as survey data was used.

2. Lines 226-231 - Statistical analysis: The statistical analysis methods section should be expanded to improve interpretability of the results.

    a. Were any of the independent variables correlated before including the variables into the models?

    b. How were the ME and AR per participant data calculated in the S2 Data Supplementary File? Is the overall value calculated using number rankings for the six question for ME and five questions for AR for the five-point Likert scales? Does that mean that a higher score for ME and AR based on the questions in the S1 Table represented feeling higher levels of being overwhelmed and frustrated, and exhaustion and tedium, respectively?

This information isn't explicitly mentioned in the manuscript and would help to improve interpretability. Please add how the values in S2 Data were calculated, particularly for ME and AR, in the statistical analysis methods section.

c. Likert scale data is generally ordinal, not continuous. Please expand further and justify the use of Poisson and negative binomial models in the methods section [2].

d. Lines 226-227: The authors stated that "The dimensions of ME and AR were assessed separately, as the questions described different feelings."
Explicitly state whether two separate models were conducted for ME and AR for both Poisson and negative binomial models. This is currently unclear in the methods but is a clearer in the results.

e. Was optimism vs pessimism analysed in the models as an independent variable? In Table 2, the final predictors were pessimism vs realistic and optimism vs realistic. Please clearly state in the methods section what comparisons were made in the statistical analysis and what the reference group for each categorical variable is.

f. The estimated coefficients were exponentiated and represented in Tables 1 to 3. This should be mentioned this in the methods section of the manuscript.

g. Line 231: Reference the R packages and the versions of the R packages used to conduct the statistical analyses.

Results:

1. Lines 259-262: The results of the survey for perceived prevalence and severity have been reported descriptively for ranges and averages, and the responses of the 801 participants has been provided in the Supplementary Information.

   For the Likert scale data, participants select only one option per question, and these options are ordinal rather than continuous. Reporting averages for this data may be misleading as the intervals between options are not necessarily equal. A mean response of 6.5, 5.0, 6.9 etc. does not accurately reflect the distribution of the responses as it isn't a continuous scale. It would be more informative to create Likert plots or represent the proportion of responses in a table for each risk area. This would allow the readers to better understand the overall distribution of message fatigue and perceived risks prior to the statistical analysis and provide a stronger descriptive representation.

   Please produce Likert plots or tables for each risk area to show the proportion of participants selecting each possible answer and discuss appropriately throughout the manuscript.

Minor comments:

Introduction:

1. Line 131: "… across studies" and different diseases? Please clarify.
2. Lines 155-156: Please reference the relevant studies to this sentence as it is stated that "few studies on message fatigue have been conducted in the context of dengue." What are the results of those studies? If there are results regarding message fatigue for dengue in the literature, the focus of the introduction should firstly be on dengue, and then the other infectious diseases. Otherwise, if no studies were identified in a literature review search to the authors knowledge, please state this.

3. Lines 158-161: Please reference relevant studies relating to these sentences: "Moreover, most studies on prevention messages for infectious diseases focus on the general population regardless of their risk levels or solely on high-risk populations concerning message fatigue. Few studies have evaluated message fatigue across groups with different risk levels, such as individuals residing in various areas but exposed to the same prolonged messaging."

Materials and methods:

1. Lines 178-185 – Participants and procedure:
    a. Lines 178-179: As this study was part of a larger project, has any of the data from the online survey been published previously and is the layout of the survey available? To ensure transparency, please cite the relevant studies and add the survey to the Supplementary Information.

2. Lines 213-215: "The score of the first question was subtracted from that of the second to categorize optimistic bias into three groups: realistic (score zero), optimistic (positive score), and pessimistic (negative score)."

    Has this method been implemented by other studies for optimistic bias? Please justify the use of this method and add interpretations of realistic, optimistic and pessimistic to aid easier comprehension of the terminology in the context of this study.

Results:

1. Lines 246-250: Please report the completion rate of the survey. How many participants started the survey but didn't answer all questions and how many answered all questions?

2. Line 318: The low dengue risk results section appears to be missing an initial sentence stating the total number of participants in that area, i.e. "The region of low dengue risk had 116 participants.". Please add this sentence to be cohesive with the other two risk area sub-headings.

3. Lines 328-329: The authors stated that "For ME in the central region, due to a lower AIC, the negative binomial model was chosen (AIC = 735.75) instead of the Poisson model (AIC = 639.12) for evaluating the associations." But the Poisson model had a lower AIC this time? Please amend appropriately.

4. Table 1, Table 2 and Table 3:
    a. As the manuscript mentions "significantly higher levels" (line 286), report the p-values in the negative binomial model tables and text for all statistical results.

    b. The abbreviated words in Tables 1 to 3 should be written in full in the footnotes for Est, SE, Exp(EST) and CI.

Discussion:

1. Lines 351-354: The manuscript states: "Combining ME and AR, in the high dengue-risk region, people with an education level equal to or below high school had higher message fatigue compared to those with a higher education level (above high school)."

    If the ME and AR models were conducted separately, then this is the result for only the AR dimension/component of message fatigue and not both dimensions. Please reword this to be specific on which dimension is being discussed as it is stated that "no significant

predictors were identified for the ME dimension" (line 350) so the dimensions shouldn't be combined.

Please do the same, where appropriate, for the entire discussion.

2. Lines 355 to 357: Following on from above, please be specific on which message fatigue dimension the statistical analysis results refer to in the discussion. The dimensions were analysed separately as they refer to different, specific feelings of message fatigue.

   For instance, the sentence could include "This indicates that people in the optimism group were more likely to experience dengue-message fatigue regarding the ME dimension compared to those in the realistic group."

3. Lines 381-384: Remove the "are" from the end of the sentence in Line 384.

4. Lines 389-395: To help corroborate these sentences, it would be beneficial to state how dengue prevention messages were framed or delivered to the public in Taiwan prior to and during the availability of this survey.

5. Lines 434-446: For the limitations, the authors state that using a convenience sampling method might not accurately reflect the entire population and that questions may have been misinterpreted.
   Please address any other biases that might exist in this survey data (i.e., not including eastern Taiwan, non-response and recall bias) and how they relate to this study.

6. Lines 434-466: Are there any future research directions from this study that could be mentioned such as implementation studies? This would help to link back to the conclusions/significance mentioned in the abstract and introduction.

7. Lines 432-434: The conclusion to the discussion appears to be at the start of the limitations paragraph and the discussion ends abruptly. To ensure that the conclusion is clearly and appropriately highlighted, please relocate these sentences to a new paragraph at the end of the discussion section and add any additional concluding information.

Supplementary Information:

1. **S1 Table:** In the manuscript, it is reported that six questions are used for ME and five questions are used for AR, but only five and four questions are reported in the table for S1 Table, respectively. Please amend this appropriately.
2. **S3 Table:** The reference group for each category in the models could be noted in this table.
3. Please make sure all Supplementary Information is referenced in the manuscript in the appropriate location.

1. Sharma, A.; Minh Duc, N.T.; Luu Lam Thang, T.; Nam, N.H.; Ng, S.J.; Abbas, K.S.; Huy, N.T.; Marušić, A.; Paul, C.L.; Kwok, J.; et al. A Consensus-Based Checklist for Reporting of Survey Studies (CROSS). *J Gen Intern Med* **2021**, *36*, 3179-3187, doi:10.1007/s11606-021-06737-1.
2. Sullivan, G.M.; Artino, A.R., Jr. Analyzing and interpreting data from likert-type scales. *J Grad Med Educ* **2013**, *5*, 541-542, doi:10.4300/jgme-5-4-18.
