# Supplementary material for: Tailoring dengue health communication: Survey-based strategies to reduce message fatigue across risk areas
Source: PLoS Negl Trop Dis. 2025 May 30;19(5):e0012723. doi: 10.1371/journal.pntd.0012723 (PMC12148230; doi:10.1371/journal.pntd.0012723)
Supplement: Attachment — Submitted filename: Response to Reviewers.pdf [file pntd.0012723.s010.pdf]

Dear Editor,

We highly appreciate the constructive comments and proposed changes made by you and the two reviewers, and would like to acknowledge your time and effort.

Below, please find a point-by-point response to the comments made by the reviewers. Our response is presented using a red font.

Reviewer #1: Major comments:

Materials and Methods:
1. Was the study conducted and reported in compliance with the Consensus -Based Checklist for Reporting of Survey Studies (CROSS) guidelines? [1]. This checklist should be completed and added to the Supplementary Information as survey data was used.
Thank you. CROSS has been provided in S6.

2. Lines 226-231 - Statistical analysis: The statistical analysis methods section should be expanded to improve interpretability of the results.

a. Were any of the independent variables correlated before including the variables into the models?
Thank you for raising this question. To assess correlations among the independent variables before including them in the models, we have conducted a Variance Inflation Factor (VIF) analysis for the full models across different risk areas. Since the full models include categorical variables, we used the Generalized VIF (GVIF) to assess multicollinearity. Based on the study by Fox and Monette [1], an adjusted GVIF (i.e., GVIF^(1/(2*Df))) value of less than two indicates no multicollinearity concerns. We have provided this explanation and the reference in Materials and methods section under the subheading statistical analysis.

As shown in S4 table, the adjusted GVIF values for the independent variables in each area are less than 2, indicating no multicollinearity issues in any of the areas. This table has been provided in the S4 Data Supplementary File. The statement regarding the multicollinearity assessment for each risk area has been included in the Results section under the subheading High dengue risk as well as Low dengue risk.

b. How were the ME and AR per participant data calculated in the S2 Data Supplementary File? Is the overall value calculated using number rankings for the six question for ME and five questions for AR for the five-point Likert scales? Does that mean that a higher score for ME and AR based on the questions in the S1 Table represented feeling higher levels of being overwhelmed and frustrated, and exhaustion and tedium, respectively?
Thank you very much for pointing out these issues. The calculation of ME and AR per participant data in the S3 Data Supplementary File was based on the sum of number rankings for the five questions in ME and four questions in AR, using a seven-point Likert scale. We have corrected the previous description, which mistakenly referred to six questions for ME, five for AR, and a five-point scale.

Additionally, a higher ME score indicates a greater sense of being overwhelmed and frustrated, while a higher AR score reflects stronger feelings of exhaustion and tedium. This interpretation aligns with the conceptual definitions in S2 Table.

We have also provided the rationale for treating Likert scale responses as count-like data and included a detailed explanation in Materials and methods section under the subheading Message fatigue.

This information isn't explicitly mentioned in the manuscript and would help to improve interpretability. Please add how the values in S2 Data were calculated, particularly for ME and AR, in the statistical analysis methods section.

Thank you for your comment. All questions were measured on a 7-point Likert scale ranging from 1 (strongly disagree) to 7 (strongly agree), with response options including strongly disagree, disagree, somewhat disagree, neither agree nor disagree, somewhat agree, and agree. For each participant, ME and AR scores were calculated by summing the numerical rankings of responses across their respective items: five questions for ME and four questions for AR, all measured on a 7-point Likert scale (S3 Table). A higher ME score indicates greater feelings of being overwhelmed and frustrated due to overexposure and redundancy of dengue messages, while a higher AR score reflects stronger feelings of exhaustion and tedium.

We have added this explanation in the Materials and methods section the subheading Message fatigue, as it directly relates to how message fatigue was conceptualized and measured. We believe this placement provides better clarity and aligns with the structure of the manuscript.

c. Likert scale data is generally ordinal, not continuous. Please expand further and justify the use of Poisson and negative binomial models in the methods section [1].
Thank you for providing the reference. We acknowledge that Likert scale data is generally ordinal, not continuous. However, our use of Poisson and Negative Binomial (NB) models is justified for several reasons. First, these models can analyze trends and associations between predictors and outcomes without relying on assumptions about precise distances between ordinal categories. Second, while Likert scale data is not strictly count data, its discrete and bounded nature resembles count data, particularly when analyzing ordinal outcomes with ordered categories. Third, when the underlying distribution of Likert scale data is skewed, raw scores tend to deviate from the true underlying distribution [2]. Therefore, treating Likert scale data as count data may better address this skewed data issue.

While ordinal logistic regression is a common approach for analyzing Likert scale data, it assumes proportional odds across categories, which may not always hold in practice [3, 4]. Additionally, it does not explicitly account for overdispersion. By contrast, NB models are specifically designed to handle overdispersion and are suited for modeling discrete, bounded, and count-like data. Poisson and NB models also focus on identifying trends and relationships without relying on the proportional odds assumption, making them more flexible for analyzing Likert responses in our context. Additionally, we compared model fit using the Akaike Information Criterion (AIC) and tested for overdispersion, selecting the NB model when the data indicated overdispersion.

For these reasons, Poisson and NB models were applied in this analysis. However, caution is needed when interpreting the results. Coefficients from models should be understood as directional trends (e.g., higher values of predictor X are associated with higher message fatigue). If coefficients are described as changes in the expected count (e.g., males experience 1.5 times higher message fatigue than females), the interpretation may not always translate well to Likert scales.

We have added the above text in the revised version under the Materials and methods section under the subheading Message fatigue and Statistical analysis.

d. Lines 226-227: The authors stated that “The dimensions of ME and AR were assessed separately, as the questions described different feelings.”
Explicitly state whether two separate models were conducted for ME and AR for both Poisson and negative binomial models. This is currently unclear in the methods but is a clearer in the results.
We have modified the text to clarify that for each dimension (Message environment and Audience response), two separate models—one Poisson and one Negative Binomial—were conducted. This explicitly states that a total of four models were run (two for ME and two for AR). We have included this clarification in the Materials and methods section under the subheading Statistical analysis.

e. Was optimism vs pessimism analysed in the models as an independent variable? In Table 2, the final predictors were pessimism vs realistic and optimism vs realistic. Please clearly state in the methods section what comparisons were made in the statistical analysis and what the reference group for each categorical variable is.
We have clarified the reference groups for all categorical variables in the revised version. For optimism bias, the realistic group was set as the reference category because it represents a neutral state, providing a baseline for comparison with the optimistic and pessimistic groups. This clarification is included in the Materials and methods section under Perceived risks.

Additionally, for demographic variables, female, higher than high school, and no job experience in dengue control were set as the reference groups for sex, education, and job, respectively. This information is now stated in the Demographic variables subsection.

Optimism vs. pessimism was analyzed as an independent variable but is not displayed in Table 2 (now Table 4 in the revised version). We believe presenting pessimism vs. realistic and optimism vs. realistic sufficiently communicates the key findings while maintaining clarity.

f. The estimated coefficients were exponentiated and represented in Tables 1 to 3. This should be mentioned this in the methods section of the manuscript.

We have clarified that the coefficients in Tables 2 and 3 are not exponentiated and represent directional trends. Additionally, we have explicitly stated that for categorical variables, coefficients are interpreted relative to the reference group, with female set as the reference for sex. This has been shown in the Materials and methods section under the subheading Statistical analysis.

g. Line 231: Reference the R packages and the versions of the R packages used to conduct the statistical analyses.
We have now included the specific versions of the R packages, specifying the versions of the MASS and car packages, along with R version 4.2.2. We have added it in the Materials and methods section under the subheading Statistical analysis in the revised manuscript.

References:
1. Sharma, A.; Minh Duc, N.T.; Luu Lam Thang, T.; Nam, N.H.; Ng, S.J.; Abbas, K.S.; Huy, N.T.; Marušić, A.; Paul, C.L.; Kwok, J.; et al. A Consensus-Based Checklist for Reporting of Survey Studies (CROSS). J Gen Intern Med 2021, 36, 3179-3187, doi:10.1007/s11606-021-06737-1.

2. Sullivan, G.M.; Artino, A.R., Jr. Analyzing and interpreting data from likert-type scales. J Grad Med Educ 2013, 5, 541-542, doi:10.4300/jgme-5-4-18.

Reviewer #2: The manuscript clearly presents the objectives of the research and the study design is appropriate in addressing the researchers' objectives. The authors gave a clear description of the study population. The statistical analysis adopted were relevant and their applications were clearly explained. Relevant ethical approval was obtained, meeting regulatory requirements for a standard scientific research.
Thank you for the comments.

Reviewer #3: Major

1. The classification of high, medium, and low risk regions is not appropriate in this article. Define southern region as a high-risk region is fine because both vectors are coexisted. However, the definition of medium and low risk regions is questionable because both regions have the same single vector. Even though the classification was based on the cumulative case number in the past, the three regions are hard to be separated equally using risk levels. Accurate definition of risk levels is important in this paper because the author want to improve the effect of public health messages in regions at various risk levels.
Thank you for your valuable feedback. We have revised the classification of risk regions in the Materials and methods section. The study now categorizes only two risk levels: a high-risk region (southern Taiwan, where both vectors coexist) and relatively low-risk regions (northern, central, eastern, and outlying islands). We have also clarified that while *Aedes aegypti* is present in some parts of the eastern region, its distribution is limited, reducing its overall contribution to transmission risk.

2. The voluntary participants might introduce selection bias. What's the age/gender/education level distribution of your study subjects? Can it comparable to the general population?
We have added a discussion in the Limitations section acknowledging potential selection bias due to voluntary participation.

3. According to the questions about either ME and AR, they are more like the feeling about the messages. From the questionnaire, it does not associate with any action changes. People who has message fatigue of dengue control does not mean they don't want to do anything to control the disease.
Thank you. We acknowledge that our study measures message fatigue as a psychological perception (ME and AR dimensions) rather than its direct effect on dengue prevention behaviors. Our analysis focuses on the associations between key predictors (age, sex, education, and perceived risks) and message fatigue across risk areas. Since we did not measure behavioral responses directly, we have clarified this limitation in the Conclusion and suggested future research to explore this relationship.

4. Regarding the "job experience (relevance to dengue control)" variable, what the reason to include this parameter? I thought you should include simply "job" information of each participant.
Thank you for your comment. We included job relevance to dengue control because individuals with experience in dengue-related work may perceive health messages differently. Prior research (Keating & Skurka, 2024) suggests that when messages are perceived as irrelevant to an individual's role, they are more likely to trigger message fatigue due to disengagement and heuristic processing. In our study, individuals working in dengue control may have higher familiarity with prevention messages**,** leading to either lower fatigue (due to alignment with their expertise) or higher

fatigue (due to overexposure). Therefore, we examined job relevance to assess how professional involvement influences message fatigue.

5. How do you treat the dependent variable in your model? Both ME and AR have multiple questions, how do you transform them in to your outcome (Y)?
Thank you for your question. ME and AR were measured using multiple Likert-scale items, and their summed scores were used as the dependent variables in our regression models. We have added explanations in Materials and methods section under the subheading Message fatigue.

Minor:
1. The demographic characteristic information of the participant can be presented as a table.
Thank you for the suggestion. We have provided the demographic characteristic in Tables 1 and 2.

Results

-Does the analysis presented match the analysis plan?

-Are the results clearly and completely presented?

-Are the figures (Tables, Images) of sufficient quality for clarity?

Reviewer #1: Major comments:

Results:
1. Lines 259-262: The results of the survey for perceived prevalence and severity have been reported descriptively for ranges and averages, and the responses of the 801 participants has been provided in the Supplementary Information. For the Likert scale data, participants select only one option per question, and these options are ordinal rather than continuous. Reporting averages for this data may be misleading as the intervals between options are not necessarily equal. A mean response of 6.5, 5.0, 6.9 etc., does not accurately reflect the distribution of the responses as it isn't a continuous scale. It would be more informative to create Likert plots or represent the proportion of responses in a table for each risk area. This would allow the readers to better understand the overall distribution of message fatigue and perceived risks prior to the statistical analysis and provide a stronger descriptive representation.
Please produce Likert plots or tables for each risk area to show the proportion of participants selecting each possible answer and discuss appropriately throughout the manuscript.
Thank you for your suggestion. We have incorporated Likert plots (Figures 1, 2, and 3) and tables (Tables 1, 2, and 3) to illustrate the proportion of responses for perceived prevalence, perceived severity, and message fatigue (ME and AR) across risk areas. These visualizations allow for a clearer representation of data distribution, avoiding misleading mean values for ordinal responses. Additionally, we have appropriately referenced these figures and tables throughout the Results and Discussion sections.

Reviewer #2: There is alignment between the analysis presented and the analysis plan. All results were well presented with clear and adequate number of tables.

Reviewer #3: Overall, the article contained two major issues. 1. Sampling scheme and small sample size make it difficult to infer to general population. 2.No clear definition of medium and low risk levels. The three models gave you different outcomes. Any novelty or insight can be inferred from those outcomes?
If you can have the information about action change of dengue control regrading message fatigue, this article will be more valuable.
Thank you for your comments. Below, we address each of your concerns:

1. **Sampling scheme and generalizability:**
   We acknowledge the limitations of our sampling scheme and sample size, which may affect the generalizability of our findings. This has been discussed in the Limitation section. In the revised manuscript, we suggest potential solutions, such as using probability sampling methods (e.g., random or stratified sampling) in future research to enhance representativeness.
2. **Risk level classification:**
   We appreciate your concern about the lack of a clear definition for medium- and low-risk levels. To improve clarity and consistency, we have revised our classification to two risk levels: high and low. This classification is

based on dengue incidence, prevalence, and vector distribution. These changes are reflected in the first paragraph of the Introduction.

3. **Differences in outcomes across risk levels:**
   Originally, we categorized risk into three levels (high, medium, low), but the medium-risk category lacked a clear epidemiological distinction. This likely contributed to variability in outcomes due to smaller sample sizes. To improve interpretability, we have consolidated medium- and low-risk into a single "low-risk" category. This adjustment provides a more stable framework for analyzing message fatigue across dengue risk areas.
4. **Novelty and insights from outcomes:**
   To address this point, we have expanded the discussion of our study's novel insights in the Conclusion section, emphasizing the implications of our findings.
5. **Dengue control action changes:**
   We agree that linking message fatigue to behavioral changes in dengue control would enhance the study's impact. However, our current analysis focuses on the associations between key predictors (age, sex, education, and perceived risks) and message fatigue across risk areas. Since behavioral responses were not directly measured, we have clarified this limitation in the Conclusion and suggested future research directions to explore this relationship.

Conclusions

-Are the conclusions supported by the data presented?

-Are the limitations of analysis clearly described?

-Do the authors discuss how these data can be helpful to advance our understanding of the topic under study?

-Is public health relevance addressed?

Reviewer #1: (No Response)

Reviewer #2: The conclusions drawn from the study are in line with the data presented by the authors and the limitations of the study were stated with sincerity. The expression of the public health relevance of the research was concise and the authors discussed how the data obtained in their study could advance understanding of the topic.
Thank you for your feedback. We appreciate your recognition of our study's conclusions, the clarity in presenting its public health relevance, and our discussion of its contributions and limitations.

Reviewer #3: Lastly, how do you apply the finding in the article to modify dengue control community education in Taiwan?
Below is a breakdown of where the study discusses applying its findings to modify dengue control community education in Taiwan, categorized by which section each recommendation appears in.

Modification	
Tailored messaging is needed instead of a uniform approach.	Discussion & Conclusion
High-risk areas: Use localized and specific messages instead of repetitive warnings.	Discussion
Low-risk areas: Messages should focus on engagement, especially for men and optimistic individuals.	Discussion
Lower-educated individuals: Use simple, relevant, and engaging messages.	Discussion
Younger audiences: Use interactive and digital content.	Discussion
Optimistic individuals: Consider loss-framed messages.	Discussion
Conduct implementation studies to test new communication strategies.	Conclusion

---

Editorial and Data Presentation Modifications?

Use this section for editorial suggestions as well as relatively minor modifications of existing data that would enhance clarity. If the only modifications needed are minor and/or editorial, you may wish to recommend "Minor Revision" or "Accept".

Reviewer #1: Minor comments:
Materials and methods:
1. Lines 178-185 – Participants and procedure:
a. Lines 178-179: As this study was part of a larger project, has any of the data from the online survey been published previously and is the layout of the survey available? To ensure transparency, please cite the relevant studies and add the survey to the Supplementary Information.
Thank you for your question. This is the first manuscript based on data from this online survey, and none of the data have been previously published. To ensure transparency, we have included the full survey layout in S1 Table, as suggested.

2. Lines 213-215: "The score of the first question was subtracted from that of the second to categorize optimistic bias into three groups: realistic (score zero), optimistic (positive score), and pessimistic (negative score)."

Has this method been implemented by other studies for optimistic bias? Please justify the use of this method and add interpretations of realistic, optimistic and pessimistic to aid easier comprehension of the terminology in the context of this study.
Thank you for raising this question. The method of calculating optimistic bias by subtracting the self-risk estimate from the perceived risk for others has been implemented in several studies [5-7].

While optimistic bias is commonly assessed using direct or indirect comparisons, our study follows the indirect comparison approach, where respondents separately estimate their own risk and the risk for others. A higher positive score indicates a stronger belief that one's personal risk is lower than that of others.

Although categorization into three groups (realistic, optimistic, pessimistic) has not been widely used, Bränström applied a similar approach, categorizing responses on a scale ranging from "Much higher than for other people" (-2) to "The same as for other people" (0) to "Much lower than for other people" (+2) [6]. This supports the validity of our classification method, as it aligns with existing frameworks for comparative risk perception.

To clarify, our study defines the three categories as follows:

- Realistic (score = 0): Respondents believe they have an equal likelihood of contracting dengue as others.
- Optimistic (score > 0): Respondents believe they are less likely to get dengue compared to others.
- Pessimistic (score < 0): Respondents believe they are more likely to get dengue compared to others.

We have included this justification and detailed category definitions in the Materials and methods section under the subheading Perceived risks.

Results:
1. Lines 246-250: Please report the completion rate of the survey. How many participants started the survey but didn't answer all questions and how many answered all questions?
The revised text, "A total of 818 individuals started and completed the survey, providing valid responses, resulting in a 100% completion rate," has been included in the Results section.

2. Line 318: The low dengue risk results section appears to be missing an initial sentence stating the total number of participants in that area, i.e. "The region of low dengue risk had 116 participants.". Please add this sentence to be cohesive with the other two risk area sub-headings.
Thank you. In the revised version, the statement "There were 348 participants in the low dengue risk region." has been included in the Results section under the subheading low dengue risk.

3. Lines 328-329: The authors stated that "For ME in the central region, due to a lower AIC, the negative binomial model was chosen (AIC = 735.75) instead of the Poisson model (AIC = 639.12) for evaluating the associations." But the Poisson model had a lower AIC this time? Please amend appropriately.
We have revised the version to include only high- and low-risk regions; the central region is no longer included.

4. Table 1, Table 2 and Table 3:
a. As the manuscript mentions "significantly higher levels" (line 286), report the p-values in the negative binomial model tables and text for all statistical results.
We have provided p-values for all statistical results in the negative binomial model tables and text

b. The abbreviated words in Tables 1 to 3 should be written in full in the footnotes for Est, SE, Exp(EST) and CI.
In the revised tables, the footnotes have been clarified, with abbreviated words written in full.

Discussion:
1. Lines 351-354: The manuscript states: "Combining ME and AR, in the high dengue-risk region, people with an education level equal to or below high school had higher message fatigue compared to those with a higher education level (above high school)."

If the ME and AR models were conducted separately, then this is the result for only the AR dimension/component of message fatigue and not both dimensions. Please reword this to be specific on which dimension is being discussed as it is stated that "no significant predictors were identified for the ME dimension" (line 350) so the dimensions shouldn't be combined.

Please do the same, where appropriate, for the entire discussion.
Thank you. We have revised the text as suggested.

2. Lines 355 to 357: Following on from above, please be specific on which message fatigue dimension the statistical analysis results refer to in the discussion. The dimensions were analysed separately as they refer to different, specific feelings of message fatigue.

For instance, the sentence could include "This indicates that people in the optimism group were more likely to experience dengue-message fatigue regarding the ME dimension compared to those in the realistic group."
Thank you for your suggestion. We have revised the discussion to ensure that each paragraph explicitly specifies which message fatigue dimension (ME or AR) the statistical analysis refers to. In the revised discussion, we have clarified the relevant dimension where applicable.
For example, we now state:
"Individuals with lower education levels experienced higher dengue message fatigue in the AR dimension in high-risk regions and in the ME dimension in low-risk regions in this study (Tables 3 and 4)."

3. Lines 381-384: Remove the "are" from the end of the sentence in Line 384.
We have removed the "are" from the end of the sentence.

4. Lines 389-395: To help corroborate these sentences, it would be beneficial to state how dengue prevention messages were framed or delivered to the public in Taiwan prior to and during the availability of this survey.
Thank you for your suggestion. We have clarified that dengue prevention messages in Taiwan were primarily delivered through government announcements, television, radio, printed materials, and social media campaigns. Additionally, we specify that these messages were framed using authoritative and instructional language, focusing on mosquito control, personal protection, and community-wide prevention efforts. These clarifications have been incorporated into the Discussion section.

5. Lines 434-446: For the limitations, the authors state that using a convenience sampling method might not accurately reflect the entire population and that questions may have been misinterpreted.
Please address any other biases that might exist in this survey data (i.e., not including eastern Taiwan, non-response and recall bias) and how they relate to this study.
Thank you for your consideration. We acknowledge the importance of addressing recall bias, and we have included a discussion of it in the limitations section of the revised manuscript.
Additionally, there were no non-responses in this study, as all participants who started the survey completed it. Details on this have been provided in the results section.
Furthermore, we categorized regions as high- or low-risk, with low-risk regions including Northern, Central, Eastern, and Outlying island regions. Therefore, Eastern Taiwan was included in the study, and we have clarified this in the revised manuscript.

6. Lines 434-466: Are there any future research directions from this study that could be mentioned such as implementation studies? This would help to link back to the conclusions/significance mentioned in the abstract and introduction.
Thank you for your feedback. We have revised the conclusion to explicitly include implementation studies as a future research direction. Specifically, we now suggest implementation studies to evaluate how different communication strategies can be applied in real-world public health settings to reduce message fatigue and enhance dengue prevention efforts.

Additionally, we have included recommendations for modifying message frequency, format, and delivery platforms to sustain engagement without increasing fatigue. We have also clarified the role of intervention studies in testing tailored messaging approaches based on psychological and demographic factors in both high- and low-risk regions.

7. Lines 432-434: The conclusion to the discussion appears to be at the start of the limitations paragraph and the discussion ends abruptly. To ensure that the conclusion is clearly and appropriately highlighted, please relocate these sentences to a new paragraph at the end of the discussion section and add any additional concluding information.
Thank you for your feedback. We have relocated the conclusion from the limitations section in the conclusion section. Additionally, we have expanded the conclusion to better summarize key findings, emphasize their significance, and provide limitations in the limitation section.

Supplementary Information:
1. S1 Table: In the manuscript, it is reported that six questions are used for ME and five questions are used for AR, but only five and four questions are reported in the table for S1 Table, respectively. Please amend this appropriately.
Thank you for your comment. S1 Table is now S2 Table. We have reviewed S2 Table and confirmed that it accurately reflects the number of questions used for ME and AR. However, we identified an inconsistency in the manuscript text and have corrected the description in the Materials and methods section to accurately report the number of questions used for ME and AR.

2. S3 Table: The reference group for each category in the models could be noted in this table.
We have made the modifications as suggested.

3. Please make sure all Supplementary Information is referenced in the manuscript in the appropriate location.
Thank you for your feedback. We have carefully reviewed the manuscript and ensured that all Supplementary Information is properly referenced in the appropriate locations.

Reviewer #2: Only minor modifications are required as indicated in the attached reviewed manuscript.
Thank you. We have reviewed and revised the text.

Reviewer #3: NA

Summary and General Comments

Use this section to provide overall comments, discuss strengths/weaknesses of the study, novelty, significance, general execution and scholarship. You may also include additional comments for the author, including concerns about dual publication, research ethics, or publication ethics. If requesting major revision, please articulate the new experiments that are needed.
Reviewer #1: This manuscript identified distinct predictors of message fatigue across different dengue-risk areas in Taiwan, including high, medium and low risk areas using survey data. The survey count data for message environment (ME) and audience response (AR) was analysed using both Poisson and negative binomial models. All negative binomial models were selected as the final output reporting only significant factors in this manuscript.

I would like to acknowledge the considerable effort that has gone into this manuscript. Understanding different factors that play a role in message fatigue including demographic factors and perception risks is highly important. As the authors state in the introduction, the knowledge of message fatigue for dengue risk in Taiwan is sparse. These findings may aid in informing updated prevention messages with future targeted implementation research specific to high, medium and low risk areas to encourage public compliance.

As the statistical analysis forms the major component of this study, the analysis should be further technically expanded in the materials and methods section to understand how the Likert scale data was treated. Also, displaying the distribution of the Likert scale data for each risk area using Likert plots or tables would greatly aid in improving this study. The Likert plots or tables would allow readers to clearly understand the overall distribution of the participants responses for message fatigue and perceived risk prior to the statistical analysis. Expanding on the above will significantly strengthen this manuscript.
Thank you for the feedback on this manuscript. In the revised version, we have explained that the Likert scale data were treated using Poisson and NB models, displayed the distribution of the Likert scale data for each risk area using Likert plots, and discussed these findings.

Reviewer #2: The research is a very unique one as there is paucity of peer reviewed articles on message fatigue regarding dengue fever. The topic is clear, the introduction is detailed and the entire manuscript is well structured and interesting to read. I strongly believe readers will also find it interesting and easy to comprehend. The research is of scientific and public health relevance as it provides insights for strategic and more community targeted preventive measures which would advance the goal of eliminating dengue fever in the study areas. I commend the efforts of the authors for this contribution to science and public health advancement
Thank you for the feedback on this manuscript.

Reviewer #3: The manuscript entitled "Different predictors of message fatigue across diverse risk areas for dengue prevention in Taiwan" evaluated message fatigue of dengue education by EM and AR related questionnaire. Although the issue is worth to investigate, the study design and the content of the questionnaire make the results less insightful.
Thank you for your feedback on this manuscript. We had already acknowledged the potential limitations of the study design in the original version and addressed them in the Limitations section.

Reference

1. Collaborators GDaH. Global, regional, and national disability-adjusted life-years (DALYs) for 333 diseases and injuries and healthy life expectancy (HALE) for 195 countries and territories, 1990-2016: a systematic analysis for the Global Burden of Disease Study 2016. Lancet. 2017;390(10100):1260-344. doi: S0140-6736(17)32130-X [pii];10.1016/S0140-6736(17)32130-X [doi].
2. Wu H, Leung S-O. Can Likert scales be treated as interval scales?—A simulation study. Journal of social service research. 2017;43(4):527-32.
3. Dolgun A, Saraçbaşı O. Assessing proportionality assumption in the adjacent category logistic regression model. Statistics and Its Interface. 2014;7:275-95.
4. Verkuilen J. A Review of Analysis of Ordinal Categorical Data, Second Edition. Journal of Educational and Behavioral Statistics. 2010;35(5):603-4. doi: 10.3102/1076998610385405.
5. Lu HY, Andrews JE, Hou HY. Optimistic bias, information seeking and intention to undergo prostate cancer screening: A Taiwan study on male adults. J Mens Health. 2009;6(3):183-90. Epub 2009/09/01. doi: 10.1016/j.jomh.2009.05.004. PubMed PMID: 32288880; PubMed Central PMCID: PMCPMC7102828.
6. Bränström R, Kristjansson S, Ullén H. Risk perception, optimistic bias, and readiness to change sun related behaviour. European Journal of Public Health. 2005;16(5):492-7. doi: 10.1093/eurpub/cki193.
7. Weinstein ND, Klein WM. Unrealistic optimism: Present and future. Journal of Social and Clinical Psychology. 1996;15(1):1-8. doi: 10.1521/jscp.1996.15.1.1.
