# Supplementary material for: Tailoring dengue health communication: Survey-based strategies to reduce message fatigue across risk areas
Source: PLoS Negl Trop Dis. 2025 May 30;19(5):e0012723. doi: 10.1371/journal.pntd.0012723 (PMC12148230; doi:10.1371/journal.pntd.0012723)
Supplement: Attachment — Submitted filename: Response to Reviewers.docx [file pntd.0012723.s011.docx]

Dear Editor,

We highly appreciate the constructive comments and proposed changes made by you and the reviewer, and would like to acknowledge your time and effort.

Below, please find a point-by-point response to the comments made by the reviewer. Our response is presented using a red font.

Reviewer #1: Minor comments:

Lines 80-82: Consider rewording to this sentence to include “where” or may be a full stop after the word pronounced.

“In high-risk regions, message fatigue is more pronounced, where repetitive warnings may contribute to disengagement, suggesting a need for communication strategies that reduce redundancy and emphasize localized, actionable information.”

Thank you for the suggestion. We have revised the sentence by placing a full stop after “more pronounced” to improve clarity.

Lines 227-229: Is it five or four questions for AR dimensions? Was one of the five questions removed? Please amend appropriately.

“For the AR dimension, the number of questions was reduced from seven to five, representing exhaustion and tedium for the same reasons (Section VI 7-11 in S1 Table), with a Cronbach’s alpha of 0.88 for the four retained questions (S2 Table).”

Sorry for the confusion. We have revised this part as “For the AR dimension, the number of questions was reduced from seven to five to represent exhaustion and tedium, due to translation-related adjustments similar to those made for the ME dimension (Section VI 7–11 in S1 Table). Of the five translated questions, one was removed during analysis because it negatively affected internal consistency. The remaining four questions yielded a Cronbach’s alpha of 0.88 (S2 Table).”

Lines 577-578: Where is this information reported in the results?

“In this study, younger individuals in low dengue-risk regions experienced higher levels of dengue message fatigue in the ME dimension.”

Thank you for pointing this out. We apologize for the confusion. The statement regarding younger individuals in low dengue-risk regions experiencing higher levels of dengue message fatigue in the ME dimension was not supported by the results. We have removed this discussion accordingly.

Figure 1: While the results section mentions that an 11-point Likert scale was used (0 = strongly disagree, 10 = strongly agree) (Lines 373-374), to ensure that Figure 1 is self-explanatory, the scale numbers (0 to 10) should be complemented with corresponding descriptors (i.e., strongly disagree to strongly agree), similar to Figures 2 and 3.

Thank you for your comment. In the revised Figure 1, we have included descriptors for scale points 0 and 10 (i.e., Not serious at all/Very unlikely and Extremely serious/Very likely) to enhance clarity. As the questionnaire did not provide descriptors for intermediate values (1–9), only the endpoints were labeled.

Figures 1-3: To ensure clarity and self-explanatory design, Figures 1, 2 and 3 should include a short, relevant title along with a labelled y-axis and x-axis.

Thank you for your suggestion. We have revised Figures 1, 2, and 3 to enhance clarity and self-explanatory presentation. Specifically, we:

- Added concise and relevant titles to the top of each figure;
- Clearly labeled the x-axis and y-axis in all figures.

Revised figures have been included in the updated manuscript.

Suggestions:

Lines 577-605: Since only high and low risk areas were evaluated and age isn’t a significant factor anymore, please consider revising this paragraph to be more concise while retaining key information about the potential need to adjust how dengue risk is communicated to the public.

Thank you for the comment. As age is no longer a significant factor in our analysis and only high- and low-risk areas were evaluated, we have removed this paragraph to maintain conciseness and clarity.

Lines 702-706: Consider rewording this sentence to include the below because public health authorities can develop different, audience-centred communication approaches using risk perception and message fatigue. However, without implementation studies to identify effectiveness, it remains unclear whether these approaches will improve the reception and impact of dengue prevention messages.

“By refining dengue messaging strategies based on risk perception and message fatigue patterns, public health authorities can aim to develop more effective, audience-centered communication approaches, which may improve the reception and impact of dengue prevention messages.”

Thank you for the suggestion. We have reworded the sentence as follows: “By refining dengue messaging strategies based on patterns of risk perception and message fatigue, public health authorities can develop more effective, audience-centered communication approaches. However, without implementation studies to assess their effectiveness, it remains unclear whether such approaches will ultimately improve the reception and impact of dengue prevention messages.”

Summary and General Comments:

Use this section to provide overall comments, discuss strengths/weaknesses of the study, novelty, significance, general execution and scholarship. You may also include additional comments for the author, including concerns about dual publication, research ethics, or publication ethics. If requesting major revision, please articulate the new experiments that are needed.

Reviewer #1: Thank you to the authors for their immense effort and time taken to address all of the reviewer comments.

Each question has been thoroughly addressed. The measurements of variables and statistical analysis methods sections have been expanded which have enhanced interpretability of the results. The inclusion of the Likert plots clearing indicate the responses of the participants and help bring clarity to the study.

The authors have made commendable improvements to the discussion by elaborating on the findings but maintaining conciseness within the discussion section, where possible, is advised. Overall, only a few minor changes and suggestions remain to further refine the manuscript.

Thank you for all your efforts. Your comments and suggestions have made this research more thorough. We are truly grateful and deeply appreciate your input.
